# Wellness in the invisible workforce: a pilot well-being study in black, indigenous, and people of color (BIPOC) women faculty in the pharmacy and pharmaceutical sciences

**DOI:** 10.1186/s12909-025-07183-x

**Published:** 2025-05-08

**Authors:** Suzanne C. Harris, Matthew Shane Loop, Kaniz Afroz Tanni, Klarissa D. Jackson, Carla Y. White

**Affiliations:** 1 Division of Practice Advancement and Clinical Education, UNC Eshelman School of Pharmacy, University of North Carolina at Chapel Hill, Chapel Hill, North Carolina United States of America; 2 Department of Health Outcomes Research and Policy, Harrison College of Pharmacy, Auburn University, Auburn, Alabama United States of America; 3 Division of Pharmacotherapy and Experimental Therapeutics, UNC Eshelman School of Pharmacy, University of North Carolina at Chapel Hill, Chapel Hill, North Carolina United States of America; 4 Dean of Culture and Well-being, Division of Practice Advancement and Clinical Education, UNC Eshelman School of Pharmacy, University of North Carolina, Chapel Hill, North Carolina, United States of America

**Keywords:** Well-being, Burnout, Pharmacy, Pharmaceutical sciences, Faculty, Mentorship

## Abstract

**Background:**

Black, Indigenous, and other People of Color (BIPOC) women faculty are underrepresented in biomedical sciences and higher education. This disparity has been highlighted in previous studies to harm productivity, career progression, and well-being. This pilot study aimed to assess the perceived impact of a longitudinal well-being program for BIPOC women faculty, estimating its effects on well-being, burnout, and self-efficacy.

**Methods:**

Full-time faculty in pharmacy or pharmaceutical science, identifying as BIPOC women, participated in this pilot repeated cross-sectional study of participants in a Well-Being Initiative, with the first cross-sectional study coming before a pilot intervention and the second cross-sectional coming after the pilot intervention. Cohort 1 engaged in a two-year program, while Cohort 2 participated for one year. Open-text questions assessed program impact on well-being, and inductive coding identified themes. Well-being, burnout, and self-efficacy were measured using the General Well-Being Index (WBI), Maslach Areas of Worklife Survey (AWS), 2-items from the Maslach Burnout Index-Human Services Survey (MBI-HSS), and General Self-Efficacy Survey (GSES). Descriptive statistics were calculated for primary and secondary outcomes.

**Results:**

Sixteen participated in Cohort 1, and 18 participated in Cohort 2. Both cohorts reported a positive impact on well-being and a sense of community as a result of the program. Post-intervention median WBI decreased, and burnout and well-being risk declined (MBI: 24% and 28% point decrease at risk for burnout, WBI: median score 4 to 3 with 20% decreased risk for poor well-being, and median score 3 to 0.5 with a 61% point decrease at risk for poor well-being, for Cohorts 1 and 2, respectively). The AWS community subscale (Cohort 1) median score increased from 2.67 to 3.5, and values subscale (Cohort 2) median score increased 3.17 to 3.67, the largest increases. Cohort 2 exhibited improved self-efficacy (GSES median score change of 30 to 34). Participants reported the sense of community and connection built over the year(s) of the program, the ability to share of experiences from those further along in their career, having a safe space to be authentic with fellow participants, and the various sessions on creating self-care plans and maintaining core values as top themes of how program most benefited their well-being. Lessons learned and opportunities for improvement are addressed.

**Conclusion:**

Participants reported many of the key impacts were the program’s ability to foster a sense of community and help them develop skills for personal and professional well-being. While higher baseline risks of poor well-being and burnout persist for BIPOC women faculty, positive trends emerged post-intervention. This study contributes to innovative strategies focused on supporting the well-being of BIPOC women faculty and lessons learned may inform and help refine future research.

**Clinical trial number:**

Not applicable

**Supplementary Information:**

The online version contains supplementary material available at 10.1186/s12909-025-07183-x.

## Background

Black, Indigenous, and other People of Color (BIPOC), with POC defined as a person who is of a race other than white [1], women faculty are disproportionally underrepresented in the biomedical sciences, and higher education at large [2]. This underrepresentation has been linked to systemic failures, such as inequitable hiring and promotion processes, salary differences, and expectations for additional service commitments including diversity, equity, and inclusion (DEI) initiatives that take time away to invest in teaching and research [3]. Similar systemic inequities have been reported in women faculty in pharmacy, such as salary inequities; imbalances in teaching, service and research; and insufficient support, including mentorship [4]. Additionally, inequities influencing the pipeline for underrepresented pharmacy faculty, such as microaggression in interviews [5], lack of access to post-graduate training [6], and lack of mentorship and advising, have also been reported [7]. In a 2024 analysis of race and gender in pharmacy faculty, disparities in career advancement was most pronounced in minority-race women, suggesting a double hurdle to be overcome [8]. These factors contribute to feelings of isolation, ^3,7^ lack of mentorship, ^7,9^ and little support for BIPOC woman faculty [3, 9]. Studies document the cumulative harms on productivity [10], career progression [8, 10, 11], and decreased well-being [3] associated with disparities in the distribution and assessment of research, teaching, and service in academia [3, 4, 10]. These occurrences have distinct implications for the hiring, promotion, retention [10], and the professional engagement [12] of BIPOC women faculty.

The National Center of Education 2021 Statistics estimates that 73% of all faculty are Non-Hispanic White, 25% are from BIPOC backgrounds, and 12% are BIPOC women of the 1.5 million faculty including professors, associate professors, assistant professors, instructors, lecturers, adjunct professors, and interim professors at degree-granting postsecondary institutions [2]. Specifically within academic pharmacy between 2023 and 2024, 63% of all faculty were White, 32% were from BIPOC backgrounds, and 14% were BIPOC women [13]. In comparison to the 2024 national census data, the proportion of Non-Hispanic White faculty overrepresented those identifying as Non-Hispanic White (58.4%) in the United States (US) population, but the proportion of BIPOC individuals (41.2%) and estimated BIPOC women (20.7%) in the US population indicates BIPOC faculty and BIPOC women faculty are underrepresented compared to the national data of similar groups [14]. Aforementioned systemic failures negatively impacting the well-being and professional advancement of BIPOC faculty also negatively influence the ability to attract and maintain a pipeline of BIPOC women faculty, further contributing to their underrepresentation in academia [10]. 

This disparity of representation of women [15] and BIPOC women extends into senior leadership positions [16, 17], and this underrepresentation is also observed in the professional advancement for BIPOC women faculty at all stages of career development [16, 17]. These trends in underrepresentation in leadership is of concern because sense of belonging [18] and fostering equity are critical aspects of well-being in pharmacy education [19]. A negative sense of belonging among BIPOC women faculty may occur from a lack of representation in leadership because this can result in fewer role models in advanced positions who understand the unique experiences of underrepresented groups [4] or a perceived lack of commitment to diversity, equity, and inclusion within a program claiming to support their diverse needs [8]. Also, if BIPOC women faculty are not ensured equitable practices, including opportunities and support for career advancement to senior leadership roles, this ultimately negatively impacts their individual success, job satisfaction, and well-being [19] According to the American Council of Education report of college presidents in 2022, men outnumber women two-to-one and 46% were White men [20]. Additionally, only 18% of US medical school deans identified as underrepresented minorities (URM),^17^ 56% of all medical school department chairs identified as White men [21]. Specifically among pharmacy faculty, 62% of department chairs were men (race not reported) [13], and White men were the most likely to advance in rank [8] or attain Chief Executive Officer dean leadership positions [8, 13], and females and racially minoritized faculty continue to experience lower rates of promotion, leadership advancement, and wages [8]. These disparities in academic leadership positions often held by White males are a concern because they may not understand the unique challenges of navigating academic career progression as a BIPOC woman [3]. In addition to the rigors of academic life, BIPOC women faculty may face racial antagonism [22], which can wear at their mental health and decrease well-being. These recurring events can result in racial fatigue, chronic stress, and burnout, leading to decreased job satisfaction and increased risk of faculty turnover [10]. 

Equally important, Lin and colleagues state that BIPOC women faculty are not a monolith, and have different expectations, interests, and experiences; however, BIPOC women faculty have had some similar collective experiences in academia that need attention, despite not being a monolith [3]. According to Chiang et al., [23] individuals with two marginalized identities are more likely to have negative mental health outcomes due to the cumulative effects of prejudice. Ultimately, meaningful change is needed to improve the representation of BIPOC women and develop a culturally inclusive and supportive professional environment to mitigate these adverse outcomes [3]. Programmatic efforts encompassing psychosocial and environmental factors should be considered to support the long-term sustainability of BIPOC women faculty. Climate and workplace burnout have been identified as key psychosocial factors that warrant further emphasis and investigation as a strategy for the retention and advancement of those in the pharmacy profession [24]. Thus, new and innovative approaches as part of programmatic efforts to enhance professional networking are needed to support the well-being, retention, and professional advancement of BIPOC women faculty [3, 16]. 

Aligned with these needs, a focus on improving well-being and decreasing burnout has been identified by key stakeholders in the pharmacy profession as a critical strategy to sustain the pharmacy workforce. Rates of burnout ranged from 41^25^-59%^26^ in pharmacy faculty, and rates of poor well-being ranged from 28^27^-37%^28^ in pharmacy professionals. However, studies assessing rates by race/ethnicity in pharmacy faculty is lacking. The American Pharmacists Association (APhA) Enhanced Well-being and Resilience National Consensus Conference called for professional associations and pharmacy programs to research well-being within the pharmacy community, evaluate aspects leading to burnout and thus decreased well-being, and identify best practices [25]. Recommendations for improving well-being and decreasing burnout in faculty include: creating a positive culture of collaboration [26, 27], promoting transparency in expectations; [26, 27] prioritizing goals and setting boundaries to improve work-life balance; [27] and institutional strategies such as flexibility in work schedules [26, 27]. However, studies on the impact of well-being strategies based on race, ethnicity, or gender is lacking. Additionally, following the consensus guidelines, the COVID-19 pandemic and heightened awareness of civil unrest were emerging, both disproportionately affecting marginalized groups [28]. BIPOC women, including those in academia, were also more likely to take on additional responsibilities, including managing difficult conversations around racial justice and the social unrest with students, colleagues, and their own children, as well as supporting their children’s remote education [3, 29]. These hardships affected people both personally [28] and professionally [3], resulting in a critical need to support the well-being of those most vulnerable to the isolation, stress, and negative impact of these ongoing events [3, 28]. 

Responding to these calls to action, we conducted a pilot study with the primary aim of determining the perceived impact of a longitudinal program focused on connection, coaching, and building community in BIPOC women faculty across multiple academic institutions of pharmacy and pharmaceutical sciences. The secondary purpose was to provide pilot estimates of the intervention’s effect on participants’ overall well-being, burnout, and self-efficacy.

## Methods

### Study design

We conducted a repeated cross-sectional study of participants in a Well-BeingInitiative, with the first cross-sectional study coming before a pilot intervention and the second cross-sectional coming after the pilot intervention. We are using the term “repeated cross-sectional” as opposed to “longitudinal” because operational study issues and dropout prevented us from reliably identifying each participant’s pre- and post-intervention outcomes. Therefore, we took the more scientifically conservative approach of by describing the study design as conducting two cross-sectional analyses of a specific population (i.e., participants in the study).

### Participants and recruitment

Approval was obtained from the University of North Carolina (UNC) at Chapel Hill and Auburn University Institutional Review (IRB) Boards. An initial cohort of BIPOC women faculty at the Assistant and Associate Professor levels (including tenure-track and research/clinical-track faculty) were recruited to participate in the program on a voluntary, first-come, first-served basis from 30 Schools of Pharmacy and Departments of Pharmacology at predominately white institutions (PWIs) from across the United States that were a mix of research intensive and non-research-intensive colleges and universities, with PWI commonly identified in scholarship as a university where 50% or more of the students are white [30]. The UNC Eshelman School of Pharmacy Marketing and Communications team assisted with disseminating IRB-approved recruitment materials directly to Schools of Pharmacy and Departments of Pharmacology via online platforms. A webpage microsite was developed to facilitate online registration for the program. Interested faculty were invited to register for the program online.

### Program overview

The Well-Being Initiative was designed to support the well-being and professional advancement of BIPOC women faculty at research-intensive universities in schools of pharmacy and departments of pharmacology and pharmaceutical sciences. The longitudinal program used a three-fold approach to well-being support for participants: (1) connection through virtual and in-person conferences; (2) coaching through ongoing wellness coaching; and (3) community through facilitating communication, professional networking, relationships, and exchange of information through an online platform. Conferences featured guest speakers, small group discussions, and self-reflective activities. The program curriculum was divided into four core units that were explored with cohort members during quarterly conferences in Years 1 and 2. The topics were: (1) Unit 1– Renew: Selfcare and Leadership; (2) Unit 2– Restore: Authenticity and Self-Advocacy; (3) Unit 3– Reset: Resilience and Negotiating Skills; and (4) Unit 4– Refocus: Goal Setting and Negotiating Advanced Roles. Conference programs were designed and developed by the vendor Houston Wellness Workshops for Women (H3W) in collaboration with faculty investigators from the UNC Eshelman School of Pharmacy. H3W implemented and facilitated the conferences and conference communications. Cohort members also had the opportunity to participate in voluntary one-on-one wellness coaching sessions with H3W offered every three months. Wellness coaching sessions were scheduled directly with H3W and the cohort member participant. Cohort members were also invited to join an online private LinkedIn group to facilitate communication and community building.

In addition to facilitating intentional connection and community between BIPOC women faculty, coaching was key to the innovative approach employed by this program. Coaching has been combined with mentoring in other programs designed to increase the interest, retention, and advancement of biomedical scientists from underrepresented minoritized groups; in this context, coaching focused on individual talent development to complement or fill in the gaps of traditional mentoring [31]. A coaching-based leadership intervention program applied to groups of business executives and middle managers was shown to increase the participants’ leadership skills as well as their self-efficacy, optimism, and resilience [32]. A systematic review of the literature on coaching as a developmental intervention suggests an overall positive link between coaching and the coachee’s self-efficacy, resilience, well-being, and goal-attainment [33]. 

#### Year 1

Recruitment for Cohort 1 of the study began in February 2021 and ended in March 2021. Twenty-three individuals completed the online registration. The study investigators reviewed the registration list, and program welcome letters were emailed to prospective participants. The welcome letter included an overview of the program, including virtual conference dates and themes. During Year 1 of the program, restrictions for travel during the pandemic limited conferences to be offered virtually only and were held quarterly: session 1 (April 2021); session 2 (June 2021); session 3 (September 2021); session 4 (November 2021). Each virtual conference met via Zoom for 1.5 days (Friday evening– Saturday).

#### Year 2

During Year 2 of the program, a second cohort (Cohort 2) of BIPOC women faculty from 30 Schools of Pharmacy and Departments of Pharmacology of similar criteria as Cohort 1 was invited to join the existing cohort to participate in program. Recruitment for Cohort 2 was conducted in January 2022. Twenty-five individuals completed the online registration. As in Year 1, study investigators reviewed the registration list, and program welcome letters were emailed to prospective participants. During Year 2, conference sessions included participants from Cohorts 1 and 2. Three virtual conferences were held via Zoom for 1.5 days (Friday evening– Saturday): session 1 (February 2022); session 2 (May 2022); session 4 (November 2022). For session 3, an in-person conference was held in September 2022 (Friday– Sunday) at a wellness retreat location. The in-person conference provided an opportunity for cohort members to meet and network, engage in personal and professional development sessions, and participate in voluntary relaxation activities in a neutral environment.

### Evaluation outcomes

The primary outcomes evaluated in the study were the perceived impact of the program on the participants’ well-being, burnout, and their self-perceptions of self-efficacy in their professional academic roles. Secondary outcomes were participants’ self-reported indicators of career advancement as well as retention within their academic institution or movement to a new institution.

### Data collection

Participants provided informed consent prior to completing the voluntary confidential well-being assessments through QualtricsXM. For the primary outcomes, the perceived impact of the program on well-being was determined by two open-text questions that were administered as the final assessment at the end of Year 2 for both cohorts. Members of both cohorts also participated in pre- and post-online assessments evaluating three factors: well-being (personal and professional), burnout, and their self-perceptions of self-efficacy in their professional academic roles. Surveys utilized validated instruments: (1) Well-being: The General Well-Being Index (WBI) for US Workers measures personal well-being and multiple dimensions of distress, in which a score ≥ 2 was used to measure personal well-being and risk of distress based on previously reported thresholds [34, 35]; (2) The Maslach Areas of Worklife Survey (AWS) measures professional well-being through employees’ perceptions of work-setting qualities. Subscale scores range 1 to 5, with 1 indicating a mismatch between person and work, 5 indicating a strong match between person and work [36]; (3) Burnout: Two single-items [37, 38] from the emotional exhaustion (“How often do you feel burned out from your work?”) and depersonalization (“How often do you feel you’ve become more callous toward people since you took this job?”) domains of the full Maslach Burnout Index-Human Services Survey (MBI-HSS) [39] were used, as these 2 items previously demonstrated to sufficiently serve as an alternative burnout assessment in health professionals with the advantage of reduced responder burden of survey length [37, 38]; and (4) the General Self-Efficacy Survey (GSES) measures self-efficacy, was adapted from the socio-cognitive theory of perceived self-efficacy according to Bandura [40], with sum scores ranging from 10 to 40, and higher scores indicate more self-efficacy. For inventories that are publicly available, previously published, or developed by authors, the survey items are available in a supplemental file. (ADDITIONAL FILE 1 Survey Items). Approval for licenses to administer proprietary instruments were obtained from the Mayo Clinic for the WBI and Mind Garden for the MBI-HSS (adapted 2 item) and MBI-AWS, though not included as full instruments in the supplemental file in accordance with the licenses.

As a secondary outcome, four questions added to the GSES surveys allowed participants to self-report indicators of career advancement and professional accomplishments (e.g., faculty reappointment, promotion and/or tenure, appointments to leadership positions, publications, awards, etc.), as well as retention within their academic institution or movement to a new institution.

Cohort 1 had the pre- and post-surveys administered to them in a staggered format over the two years of their program. The timeline for Cohort 1 pre-surveys was: (1) session 1 (April 2021): WBI and AWS, (2) session 2 (June 2021): GSES and career advancement, and (3) session 3 (September 2021): MBI. The timeline for Cohort 1 post-surveys during Year 2 was: (1) session 1 (February 2022): WBI and AWS, (2) session 2 (May 2022): GSES and career advancement, (3) session 3 (September 2022): MBI, and (4) session 4 (November 2022): two open-text questions on the perceived impact of the program.

Cohort 2 had the pre- and post-surveys administered to them in a combined assessment format over the one year of their program. The Cohort 2 pre-surveys were administered in February 2022, and included the WBI, AWS, GSES and career advancement, and MBI assessments. The Cohort 2 post-surveys were conducted during their fourth and final session in November 2022 and included the same assessments as the combined pre-survey plus two open-ended questions on the perceived impact of the program.

### Data analysis

Open-text responses were analyzed using thematic coding completed by two study personnel using a constant comparative approach. A codebook was developed using inductive coding techniques and applied to the survey responses by one study personnel. A second investigator served as an auditor to review the codebook and coded themes for agreement as well as to identify any emerging themes. Any discrepancies were discussed to reach a consensus.

Data analysis of assessments compared pre- and post-survey responses, both within cohorts and between cohorts, to identify the impact of the program on participants’ well-being, burnout, and self-efficacy over time. Because this was a pilot study, we did not conduct null hypothesis significance testing to detect efficacy of the intervention. Rather, we presented descriptive estimates of the change in outcomes from pre- to post-intervention. Summary statistics were calculated for demographic variables and all primary and secondary outcomes. Continuous variables were summarized using the median (minimum, maximum). The median was chosen as the measure of central tendency, as opposed to the mean, because not all of the instruments had clearly symmetric or monomodal distributions. The mean and median were not always clearly similar. Using the median as the measure of central tendency allowed all instruments to have a clear interpretation of where 50% of the values were above and 50% of the values were below the stated median. Categorical variables were summarized using counts (percentages). Sample statistics for demographic variables were calculated by study cohort. Summary statistics for outcome variables were calculated by study cohort and by time of assessment (i.e., pre-intervention and post-intervention). Due to the limited participants completing all assessments, each assessment for each cohort was summarized using all the study participants who completed that assessment, not only participants who completed both the pre- and post-intervention assessments for a given outcome. Therefore, the sample sizes varied among assessments. Differences in median outcome from post-intervention compared to pre-intervention were calculated for well-being and self-efficacy, while differences in proportions were used for comparing being at risk for burnout and for all secondary outcomes. The differences in medians and proportions were summarized visually using dot plots. These estimates can serve as preliminary estimates of efficacy for future, fully statistically powered studies to test the efficacy of the intervention. The R computing environment was used (R Core Team 2022), drawing heavily on the tidyverse [41] and gtsummary packages [42], to conduct the analysis.

## Results

There was a total of 44 women faculty who participated in the intervention (i.e., well-being program), 21 in Cohort 1 and 23 in Cohort 2. Intervention participant engagement was generally similar between Cohort 1 and Cohort 2 during virtual conference sessions. For example, 12 participants from Cohort 1 and 13 participants from Cohort 2 attended the first virtual conference retreat in year 2, in which concurrent cohorts were enrolled. These attendance numbers represent 52% (12 out of 23) and 62% (13 out of 21) of the original number of registrants for Cohorts 1 and 2, respectively. Intervention participant attendance was also generally consistent for virtual conference sessions in year 1 of 2, for Cohort 1. For example, the number of intervention participants who attended each of the four quarterly virtual conference retreats in year 1 was 13, 15, 12, and 13 for Cohort 1.

Not all intervention participants completed the well-being surveys. Results summarized below are from the survey participants only. Table 1 summarizes the characteristics of the two survey participant cohorts. In total, 16 BIPOC faculty participated and completed surveys in the 2-year program (Cohort 1), while another 18 participated and completed surveys in the 1-year program (Cohort 2). Among survey participants who listed their gender identity, all self-identified as a female. The pluralities of both Cohorts 1 (67%) and 2 (50%) self-identified as Black or African American, and the second most common self-identified race was Asian or Pacific Islander. Survey participants in Cohort 1 had similar proportions of Assistant Professors (*n* = 8, 53%) and Associate Professors (*n* = 7, 47%). On the other hand, the majority of survey participants in Cohort 2 were Assistant Professors (*n* = 13, 72%). In both the cohorts, approximately 20% of the survey respondents had an administrative title.

**Table 1:** Demographics of study participants who completed well-being assessments

Characteristic	Cohort 1 (*N* = 16^*1*^)	Cohort 2 (*N* = 18^*1*^)
Academic Rank		
Instructor/Lecturer	0 (0%)	0 (0%)
Assistant Professor	8 (53%)	13 (72%)
Associate Professor	7 (47%)	5 (28%)
Professor	0 (0%)	0 (0%)
Unknown	1	4
Race/ethnicity		
Asian or Pacific Islander	4 (27%)	6 (33%)
Black or African American	10 (67%)	9 (50%)
Hispanic or Latino	0 (0%)	2 (11%)
Native American or Alaskan Native	0 (0%)	0 (0%)
White or Caucasian	0 (0%)	0 (0%)
Multiracial or biracial	0 (0%)	0 (0%)
Afro-Caribbean	1 (6.7%)	0 (0%)
A race/ethnicity not selected here	0 (0%)	1 (5.6%)
Unknown	1	0
Gender identity		
Female	15 (100%)	18 (100%)
Male	0 (0%)	0 (0%)
Transgender Female	0 (0%)	0 (0%)
Transgender Male	0 (0%)	0 (0%)
Gender variant/non-conforming	0 (0%)	0 (0%)
Not listed	0 (0%)	0 (0%)
Unknown	2	4
Has administrative title	3 (20%)	4 (22%)
Unknown	1	4

^*1*^n (%)

### Qualitative data

Free-text responses to the question, “How has the Well-being Initiative for Women Faculty of Color Program influenced, positively or negatively, your well-being?” offered in-depth and personal experiences and insights on the primary outcome of survey participants’ perceived impact of the program. For survey participants in Cohort 1, five core themes were identified using thematic coding, including, in rank-order: (1) community, (2) positive impact, (3) authenticity, (4) psychological safety, (5) and empowerment to self-advocate. For survey participants in Cohort 2, two core themes were identified, including (1) community and (2) positive impact. These were also the two top themes for both cohorts. Specifically for the theme of community, one survey participant stated “[The program] absolutely and overwhelmingly positively impacted my well-being! The ways in which we’ve been able to connect over the past [two] years, build community with other ladies with shared experiences, safe spaces to be transparent, [being] more aware of how our environments either do or do not support, [and] being able to bring my full authentic self to work [were influenced by the program].’ Another emphasized the community and intentionality of the program as being beneficial, sharing “It’s given me a sense of community, belonging, support and comfort that I knew would be out there but didn’t get the opportunity to develop myself. And the intentionality of it makes it so much more invaluable.” Other survey participants spoke to specific sessions and activities of the program that most contributed positively to their well-being. One survey participant shared, “The program has added to my well-being in a positive manner. The sessions on values, personal strategic plan, and the sessions that spoke about eating healthy, etc.were the most beneficial in influencing my well-being.” Another appreciated the opportunities to connect in different formats, stating “I’ve felt every emotion during our program online session, one-on-one coaching, and our in-person retreat. This program has given me permission to rest and know that ultimately, I will thrive because of it. I don’t think any other program could’ve done these things for me in such a short period of time and that is reflective of all the wonderful women of color who led and organized all activities.”

Free-text responses to the question, “What are 2–3 strategies that you feel you are most likely to apply to foster your personal and/or professional well-being?” also offered insight into how survey participants intended to carry learned strategies forward. For Cohort 1 survey participants, three core themes were identified, including 1) prioritization, (2) negotiation, and (3) self-advocacy. For Cohort 2, they expressed similar core themes of prioritization and negotiation, though they also expressed physical reset/rest and building community as core strategies they hoped to apply. Prioritization was a top theme for survey participants in Cohort 1 and negotiation was the top theme for Cohort 2. Additional examples of free-text responses in each core theme are included in Table 2.

**Table 2:** Program impact core themes rank-ordered by prevalence

Question focus	Themes	Sample Responses
Impact of the program on participant well-being	Community	“It’s [the program] given me a sense of community, belonging, support and comfort that I knew would be out there but didn’t get the opportunity to develop myself.” “… helped me connect with new and old friends on a deeper level–forging bonds that will help me in the future.”
	Positive impact	“As a newer faculty member this program was amazing. Being able to get guidance from those who have paved the way before me, I will be forever grateful for [the program].”
	Authenticity	“I really needed this safe space to practice how to bring my authentic self to work, to be vulnerable, to be seen, to be heard, to be celebrated”
	Psychological safety	“I’ve learned that some things that I may have been struggling with were not obscene and they are true feelings.”
	Empowerment to self-advocate	“The program has given me permission to put myself first and to unapologetically take care of my health all around.”
Strategies participants reported most likely to apply	Prioritization	“I think my biggest takeaway from this program has been learning to prioritize my own self-care, but reframing it not as separate from my other ‘to do’ lists - but rather as an integral part of having the capacity to be able to do all the things”
	Negotiation	“… make demands if necessary to accommodate my successfulness in academia.” “Laying out information and data in tangible ways before negotiating with administration”
	Self-advocacy	“I am in full authority to self-advocate for what I need” “[I plan to] review my quarterly strategic plan and ensure it aligns with my core values.”
	Physical reset and rest	“… being more proactive with taking email-free vacations (realizing that I am not indispensable and the world will keep turning even if I take a break)”
	Building community	“Leveraging the community of faculty found through this cohort.” “…finding colleagues at work who are willing to be my advocates”

### Quantitative data

Because all outcomes were measured at the same time in Cohort 2, the response rate for survey participants was the same across all outcomes. For Cohort 2 survey participants, the response rate for the main outcomes (well-being, professional well-being, burnout, and self-efficacy) were 100% pre-intervention and 33% post-intervention. In Cohort 1 survey participants, the response rates varied across the main outcomes. The response rates for pre-intervention outcomes ranged from 69% for burnout to 94% for well-being. The response rates for post-intervention outcomes ranged from 38% for self-efficacy to 69% for the well-being. Response rates for post-intervention assessments for survey participants in Cohort 1 were generally much higher than for Cohort 2.

**Fig. 1:**
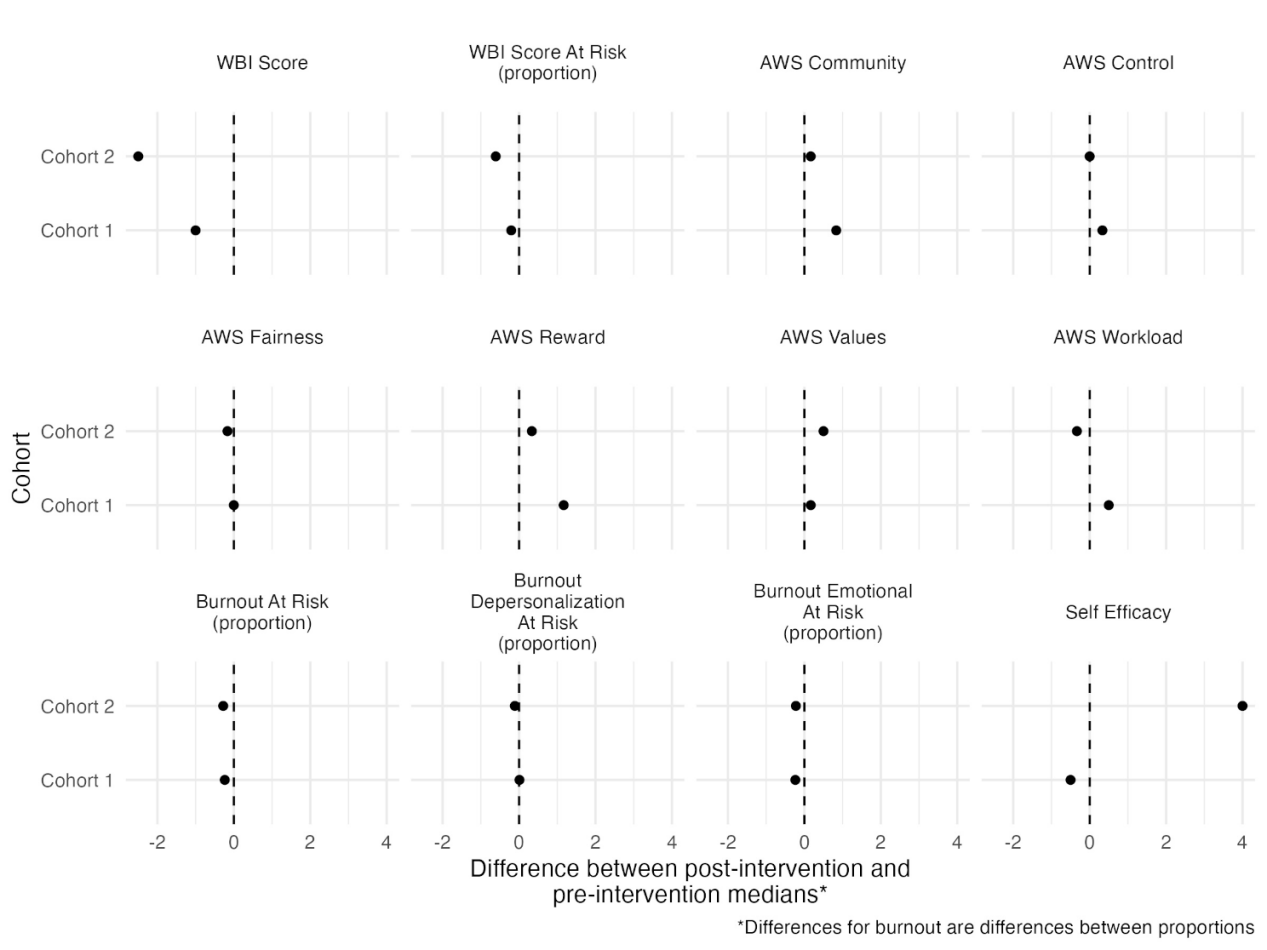
Dotplots of differences in medians (or proportions) between post- vs. pre-intervention for primary outcomes

Table 3 condenses these summary statistics into medians or proportions for the primary outcomes, as well as estimated differences from pre- to post-intervention. More detailed statistics on the primary and secondary outcomes are included in ADDITIONAL FILE 2 Table. Figure 1 displays the information in Table 3 in a visual format as dot plots. Therefore, all the results that follow from Table 3 are reflected in Fig. 1. The first primary outcome was well-being, measured by both the WBI (personal well-being) and the AWS (professional well-being) instruments. Both survey cohorts had comparable pre-intervention median WBI scores (4 for Cohort 1 and 3 for Cohort 2). From Table 3, we see that the median WBI decreased for both survey cohorts (4 to 3 for Cohort 1 and 3 to 0.5 for Cohort 2), indicating an improvement in personal well-being. Based on the binary version of WBI, ‘at risk for low well-being’, 14 (93%) survey participants in Cohort 1 and 14 (78%) survey participants in Cohort 2 were at-risk of low well-being in the pre-intervention period, which decreased to 8 (73%) in Cohort 1 and 1 (17%) in Cohort 2 in the post-intervention period. For the workplace well-being domains, survey participants in Cohort 1 experienced an increase in AWS scores in 5 of 6 subscales (workload, control, reward, community, and values), indicating improvement in professional well-being, while the AWS median score for the fairness subscale was unchanged in the post-intervention period. These findings were not as consistent in Cohort 2 survey participants. Similar to Cohort 1 survey participants, there was a positive change in the community, reward, and values subscales, while the fairness and workload scale scores decreased post-intervention, indicating lower professional well-being in these subscales. The control subscale was unchanged in Cohort 2 survey participants. In Cohort 1 survey participants, the largest increase was observed for the reward subscale, while in Cohort 2 survey participants the largest increase was seen in the values subscale.

**Table 3:** Medians and proportions of primary outcomes post- and pre-intervention, with pilot estimate of treatment effect

Cohort	Instrument	Post-intervention	Pre-intervention	Difference
Well-Being				
Cohort 1	WBI Score	3.0	4.0	**-1.0**
Cohort 2	WBI Score	0.5	3.0	**-2.5**
Cohort 1	WBI Score At Risk (proportion)	0.7	0.9	**-0.2**
Cohort 2	WBI Score At Risk (proportion)	0.2	0.8	**-0.6**
Professional Well-Being				
Cohort 1	AWS Community	3.5	2.7	**0.8**
Cohort 2	AWS Community	3.7	3.5	**0.2**
Cohort 1	AWS Control	3.3	3.0	**0.3**
Cohort 2	AWS Control	3.8	3.8	**0.0**
Cohort 1	AWS Fairness	2.3	2.3	**0.0**
Cohort 2	AWS Fairness	2.8	3.0	-0.2
Cohort 1	AWS Reward	3.8	2.7	**1.2**
Cohort 2	AWS Reward	4.0	3.7	**0.3**
Cohort 1	AWS Values	3.5	3.3	**0.2**
Cohort 2	AWS Values	3.7	3.2	**0.5**
Cohort 1	AWS Workload	2.5	2.0	**0.5**
Cohort 2	AWS Workload	2.5	2.8	-0.3
Burnout				
Cohort 1	Burnout At Risk (proportion)	0.4	0.6	**-0.2**
Cohort 2	Burnout At Risk (proportion)	0.3	0.6	**-0.3**
Cohort 1	Burnout Depersonalization At Risk (proportion)	0.1	0.1	0.0
Cohort 2	Burnout Depersonalization At Risk (proportion)	0.0	0.1	**-0.1**
Cohort 1	Burnout Emotional At Risk (proportion)	0.4	0.6	**-0.2**
Cohort 2	Burnout Emotional At Risk (proportion)	0.3	0.6	**-0.2**
Self-Efficacy				
Cohort 1	Self Efficacy	30.5	31.0	-0.5
Cohort 2	Self Efficacy	34.0	30.0	**4.0**

All statistics are medians except for the ‘at risk’ variables, which are proportions. Each assessment for each cohort was summarized using all the study participants who completed that assessment, thus sample sizes varied among assessments. Consistent with the goal of a pilot study being preliminary estimation of the possible effect size, as well as the small sample size, *p*-values or confidence intervals for the mean differences were not provided

The second primary outcome was burnout, measured using the 2-item MBI and categorized into risk of depersonalization burnout, risk of emotional burnout, and total burnout risk (at risk for either depersonalization or emotional burnout). For the emotional exhaustion domain of burnout, 64% (*n* = 7) of survey respondents in Cohort 1 and 56% (*n* = 10) in Cohort 2 were at risk during the pre-intervention stage. These percentages decreased to 40% (*n* = 4) for Cohort 1 survey participants and 33% (*n* = 2) for Cohort 2 during post-intervention, indicating decreased risk for emotional exhaustion. Of pre-intervention survey participants, 1 (9.1%) in Cohort 1 and 2 (11%) in Cohort 2 were at risk of depersonalization. This percentage remained approximately the same in Cohort 1, while it decreased to 0 (0%) survey participants in Cohort 2 in the post-intervention period, indicating no change (Cohort 1) or decreased risk (Cohort 2) of depersonalization as burnout. Finally, total burnout risk decreased in both survey participant cohorts after the program. The proportion of survey respondents at-risk of total burnout decreased from 64 to 40% in Cohort 1 and from 61 to 33% in Cohort 2.

The third primary outcome, self-efficacy, was measured using the GSES. The survey participant cohorts had similar median GSES scores pre-intervention (31 for Cohort 1 and 30 for Cohort 2 on a scale of 0–40). The post-intervention comparison shows that there was a slight decrease in the GSES median score in Cohort 1 survey participants (to 30.5) and a slight increase in the median GSES score (to 34.0), indicating improved self-efficacy scores for Cohort 2 survey participants following the intervention.

For the secondary outcomes of career advancement and professional accomplishments in the last 12 months (ADDITIONAL FILE 2 Table), the most common pre-intervention accomplishments reported by both survey participant cohorts were obtaining a faculty reappointment and being invited to speak at conferences. For the same career advancement question asked post-intervention, faculty reappointments were the most often reported by Cohort 1 survey participants and appointments to leadership positions within their institution were most often reported by Cohort 2 survey participants. In the post-intervention period, peer reviewed publications were the most often reported professional accomplishment by both survey participant cohorts. More cohort survey participants had transitioned to a new academic institution within the 12 months before the intervention compared to the 12 months leading up to the post-assessment. No survey participants in either cohort had moved to a non-academic institution within the 12 months of the pre- or post-assessment.

## Discussion

Women faculty who identify as BIPOC are underrepresented in academia [2], including in pharmacy and pharmaceutical sciences faculty [13] and disparities exist in the distribution of research, teaching, service, mentorship and support in academia, including pharmacy and pharmaceutical sciences fields [3, 4]. BIPOC women faculty also experience adverse social climates disproportionately affecting marginalized groups, which can have negative impacts on productivity, promotion, and well-being [3, 8, 28]. Despite these disparities, a gap exists concerning what interventions may beneficially impact burnout, well-being, and self-efficacy among BIPOC women faculty. This study is one of the first to pilot the perceived impact of a longitudinal well-being program, as well as assess pilot efficacy on well-being, burnout, self-efficacy, and career advancement outcomes in BIPOC women faculty. This discussion aims to synthesize the findings identified through the well-being assessments to provide insight into the effectiveness of the program on well-being, burnout, and self-efficacy, and thus ultimately inform future directions for implementing and evaluating well-being programs to support BIPOC women faculty. Outcomes on well-being, burnout, and self-efficacy that align with existing studies will be discussed, though it should be noted there is a lack of similar interventions focused on the impact of a well-being program in BIPOC women faculty with measured outcomes. Thus, comparisons to a similar intervention in the same target group will not be feasible. This study was successful in its objective of assessing perceived impact of a longitudinal well-being program focused on connection, coaching, and building community among BIPOC women faculty across multiple institutions. Additionally, the outcomes on well-being, burnout, and self-efficacy trended in a beneficial direction across most measures, though as a pilot study, the findings cannot definitively assess efficacy. Nonetheless, this study aligns with calls to action for improving faculty well-being and investing in our human capital through diversity [26, 43]. 

### Synthesis of qualitative and quantitative data on well-being, burnout, and self-efficacy outcomes

Among the core qualitative themes expressed by our survey participants related to the perceived effectiveness of the program, positive impact on their well-being and sense of community resonated equally with both cohorts. Survey participants shared aspects of the program that fostered a sense of community and positive well-being, specifically the ability to connect and maintain relationships over the 1-to-2-year program, to build a community with other BIPOC women with shared experiences that they had yet to develop in their work environments, and to have a safe space to be transparent and be their authentic selves. Other studies have explored the intersectionality of individual well-being and impact on organization [44]. Thus, implementation of well-being programming as part of a larger organizational behavior change may also be helpful to achieve powerful outcomes [45, 46]. Grum and Babnik [47] point out that well-being is an essential part of social sustainability and that high levels of well-being lead to greater performance and productivity. When organizations prioritize the well-being of their staff, it not only benefits individual employees but also contributes to the overall effectiveness and sustainability of the organization itself. Having a sense of community was also expressed by survey participants in our study, and additional studies show that social connectedness and a sense of belonging in association with meaningful engagement are important aspects of well-being and appear to be a basic human need [18, 46, 48–50]. These findings highlight that what our survey participants valued and found effective also aligns with previous literature that recommend programs should invest in infrastructure along with organizational strategies that encourage commitment and accountability to foster inclusion and social connections that positively impact mental and emotional well-being [51]. 

Personal well-being scores in this study showed that 93% and 78% (Cohort 1 and 2, respectively) of the survey participants were at risk of poor well-being at baseline, which was higher than previous reports of poor well-being in the national population and within pharmacy professionals [34, 37]. As for burnout measures, this study’s survey sample had higher baseline rates of overall burnout and the emotional exhaustion subdomain of burnout (approximately 60% across both Cohort 1 and 2 survey participants) than previous studies of burnout prevalence in the broader pharmacy faculty [37, 52, 53]. The higher baseline rates of burnout in our survey study population are consistent with the sparse reports of higher rates of burnout in women pharmacy faculty [52] and in medical school students with multiple marginalized identities (e.g., non-white and female) [54]. Despite these higher baseline rates of poor well-being and burnout, it is noteworthy that the post-intervention well-being and burnout scores improved and the percentage at risk decreased (MBI: 24% and 28% point decrease, WBI: 20% and 61% point decrease, Cohort 1 and 2 survey participants, respectively). These positive trends could be explained by the perceived impact of the program identified by the survey participants. Positive sentiments expressed by survey participants related to: (1) the design of the program over one or two years that allowed for intentional opportunities to continue to build connections; (2) the development of skills for prioritizing self-care and self-advocacy for core values, both as a group and through one-on-one coaching; and (3) availability of both online and in-person retreat for reinforcing flexibility with different avenues to stay connected. All are evidence of the perceived success of the program which likely influenced their post-intervention responses on burnout and personal well-being assessments. Based on these findings, future studies with a larger cohort within pharmacy and pharmaceutical science programs are warranted to confirm the effectiveness of these early positive findings.

When assessing professional well-being, there are even more limited studies within academia or the pharmacy profession compared to studies on burnout and personal well-being. Results from this study found that the AWS subscales of workload and fairness had the lowest scores, which is somewhat consistent with AWS measures in community pharmacists [55]. However, our survey sample had higher scores for the control subscale (i.e., indicating a strong match between person and work or higher professional well-being). While most AWS scores also improved post-intervention, this improvement was not consistent between survey participant cohorts. The decrease in post-intervention AWS fairness and workload subscales in Cohort 2 survey participants only, which indicate lower professional well-being in these areas, may be a result of Cohort 2 survey participants having a higher percentage of assistant professors compared to Cohort 1 (72% versus 53%, respectively). Junior pharmacy faculty have been reported to have higher rates of burnout [52, 56] and overwhelming workload, especially if coordinating responsibilities between practice and school sites [56]. In addition to heavy workload, a lack of autonomy to manage their time, which may be perceived as unfair or disadvantaged, has also been reported by assistant professors in pharmacy [57]. Another explanation could be related to time and experience. Cohort 2 survey participants had a shorter time in the program (1 year versus 2 years) to apply skills learned, and higher ranked faculty have more experience and time in their career to develop skills in stress management and burnout, allowing them to better navigate challenges in academia [56]. Thus, future studies are warranted to assess the impact of well-being strategies on professional well-being in pharmacy and pharmaceutical sciences faculty, including in lower rank BIPOC women faculty who are at higher risk.

Self-efficacy refers to an individual’s belief in his or her capacity to execute behaviors necessary to produce specific performance attainments [58]. The self-efficacy scores for Cohort 1 survey participants were mostly comparable to those reported in national US adult sample, though Cohort 2 survey participant scores were slightly higher, indicating greater self-efficacy [59]. Slightly more of Cohort 2 compared to Cohort 1 survey participants reported career advancements in the past 12 months on the post-intervention survey related to: (1) moving into a new academic institution, (2) changing to tenure status, (3) being appointed to leadership position within their institution, (4) obtaining a grant, and (5) receiving a faculty research or service award. (ADDITIONAL FILE 2 Table) These successes may have allowed Cohort 2 survey participants more opportunities to reflect on past successes, which reflection is a strategy for building self-efficacy [60], and may explain the differences in results. Assessing self-efficacy is important because in addition to burnout, professional self-efficacy is a factor that influences both workplace and psychosocial well-being, as well as workplace performance [61, 62]. One study in physicians found that self-efficacy was associated with a low risk of burnout [63], and another in international faculty found burnout was an antecedent of self-efficacy, underscoring the importance of efforts to address overwork and exhaustion in faculty [64]. However, self-efficacy as measured by the GSE, is not commonly reported in the pharmacy or pharmaceutical sciences literature. Thus, our study was unique in exploring this domain. Considering our survey participant cohorts’ self-efficacy scores were similar if not slightly improved over the general population, their inherent sense of self-efficacy may be a protective factor against burnout and a positive factor for workplace performance. Future research is warranted to further explore the relationship of self-efficacy not just on burnout, but also work engagement and life satisfaction, in pharmacy and pharmaceutical sciences faculty.

While this study addressed a critical gap in the literature, it had limitations. While the size of program cohorts was intentionally kept small to allow for authentic conversation and deeper dialogue, the small sample size may have impacted generalizability. Also, due to the pilot study design and the small number of participants who completed all assessments, the analysis of within-subject responses or conduct of null hypothesis significance testing for statistical differences between survey participant cohorts was not feasible. We also used a pre-post design instead of a true control group of participants who received no intervention, which limits our ability to make causal inferences. With all assessment being voluntary, the potential for survey participant self-selection bias also exists. For example, the small number of participants who completed all assessments may have been participants who had systematically better well-being, so we may have overestimated the preliminary estimates of effect in this study. This overall study also had the potential for sampling bias, as participants who enrolled may have had an interest in the topic. For example, if the survey participants are already highly satisfied or dissatisfied with their well-being, they may be more likely to complete a feedback survey, potentially skewing the results. Therefore, our results may not generalize to all BIPOC women faculty. The difference in study design between the two survey cohorts may have influenced outcomes, including the more robust changes observed for Cohort 2 versus Cohort 1 survey participants. As part of this pilot study, different assessment sequence strategies were conducted between the two survey cohorts. The staggered approach (i.e., each assessment disseminated sequentially on a designated session over the two year program) with Cohort 1 compared to the combined assessment approach (i.e., combined all pre-surveys in the first session, combined all post-surveys in the last session) for Cohort 2 may have influenced the extent of change between pre- and post-intervention measures in survey participants. It is unknown whether the extent of travel limitations due to the COVID-19 pandemic, which affected the ratio of in-person to online sessions (1:8 for Cohort 1 and 1:4 for Cohort 2), influenced responses. Lastly, it is unknown how the extent of interrelatedness between content covered across sessions affected responses on the surveys (i.e., a prior session on burnout influencing an individual’s response on the self-efficacy survey). Future studies are warranted to identify the best assessment approaches to measure impact of longitudinal well-being interventions.

### Lessons learned and opportunity for refinement

Despite limitations, this pilot study can inform future directions for implementation and evaluation of well-being programs. Some lessons learned can be carried forward to inform refinements and improvements for others who are seeking to successfully replicate and expand this program to their institutions. For recruitment, we learned it is essential to acknowledge the challenge of fitting participation into faculty’s busy schedules. Encouragement from leadership is essential and motivates them to invest in their personal and professional growth, and thus engaging leadership early was a critical first step. By leadership clearly communicating the benefits and outcomes of the program, this help participants understand the value they will gain from their involvement. This process often requires multiple outreach efforts to ensure the message is received and considered. Also, as part of a successful recruitment effort, a strategic marketing approach is essential in recruiting a target audience. For example, program participants shared similar professional environments, career advancement processes, experiences, and aspirations; and thus, could see the benefits of enrolling in a program to support people of similar needs.

Maintaining continued engagement and attendance over the 1 to 2-year program was also a challenge we faced. Identifying an audience with a common goal and need can help this. For example, participants faced similar treatment from the world, which further bonded them. This common ground allowed the participants to connect on a deeper level, provided them with a sense of validation and inspiration to maintain engagement in the program. In addition, being intentional in the design and elements of the program is important for sustained engagement. Specifically, fostering a supportive environment and providing consistent engagement through coaching and sponsorship, as well as skill and leadership development will inspire participation. Additionally, other factors may have affected participant engagement and attendance, including busy work and/or home schedules, or competing responsibilities. Online conferences were scheduled largely during weekend hours (Friday evenings-Saturday daytime) rather than during the weekdays. While the intent of this approach was to avoid work week conflicts, weekend sessions may have affected some individuals’ ability and willingness to participate in the program. Future programs could consider alternating weekday and weekend sessions as an approach to address scheduling conflicts. Other strategies to address attendance that were helpful included offering various formats of the content delivery (e.g. mix of Zoom and in-person sessions), and willingness to adapt program design based on feedback (e.g., shortened total duration during weekend sessions). These strategies can provide participants more flexibility to attend, better manage their time and work conflicts, and thus support work-life balance. Lastly, response rates on well-being surveys were low, and thus dedicating time during the program sessions for participants to complete well-being surveys in real-time may improve survey response rate. These are ideas to refine strategies that may increase the likelihood of success for institutions seeking to replicate an effective and impactful program.

## Conclusion

This study highlights a promising first step in designing a longitudinal well-being program focused on connection, coaching, and building community in BIPOC women faculty across multiple institutions. The combination of quantitative and qualitative data from the surveys provides a foundation upon which to build future studies. This study was the first of its kind to pilot the perceived impact of a longitudinal well-being program in BIPOC women faculty in pharmacy and pharmaceutical sciences. Survey participants reported many of the key impacts were the program’s ability to foster a sense of community and help them develop skills they can use to improve their personal and professional well-being. While findings suggest BIPOC women faculty may be at higher risk of poor well-being and burnout compared to the general population and the broader academic pharmacy and pharmaceutical sciences community, there were positive trends in post-intervention outcomes for well-being, burnout, and self-efficacy in survey participants. Lessons learned can inform future research which should examine the feasibility and scalability of cross-institutional, longitudinal well-being programs in a larger biomedical sciences cohort and include long-term follow-up.

## Electronic supplementary material

Below is the link to the electronic supplementary material.

**Supplementary Material 1**: **Additional file 1**: Survey Items (Pre and Post Assessments).

**Supplementary Material 2**: **Additional file 2**: Pre- and post-intervention summary statistics for primary and secondary outcomes by cohort.

## Data Availability

All data generated or analyzed during this study are included in this published article [and its supplementary information files].

## Abbreviations

BIPOC: Black, indigenous, and other people of color
WBI: General well-being index
AWS: Maslach areas of worklife survey
MBI-HSS: Maslach burnout index-human services survey
GSES: General self-efficacy survey
DEI: Diversity, equity, and inclusion
US: United States
URM: Underrepresented minorities
APhA: American pharmacists association
H3W: Houston wellness workshops for women
UNC: University of North Carolina
IRB: Institutional review board
PWI: Predominately white institutions
