# Supplementary material for: Wellness in the invisible workforce: a pilot well-being study in black, indigenous, and people of color (BIPOC) women faculty in the pharmacy and pharmaceutical sciences
Source: BMC Med Educ. 2025 May 8;25:674. doi: 10.1186/s12909-025-07183-x (PMC12060405; doi:10.1186/s12909-025-07183-x)
Supplement: Supplementary file 1 — Supplementary Material 1: Additional file 1: Survey Items (Pre and Post Assessments). [file 12909_2025_7183_MOESM1_ESM.docx]

| **Survey Items (Pre and Post Assessments)**   1. **Well-being Assessments** |
| --- |
| **Demographic** |
| Which of the following best describes you? *Please select one answer*.   1. Female 2. Male 3. Transgender Female 4. Transgender Male 5. Gender variant/Non-conforming 6. Not listed (open text)   Which of the following best describes you? *Please select one answer*.   1. Asian or Pacific Islander 2. Black or African American 3. Hispanic or Latino 4. Native American or Alaskan Native 5. White or Caucasian 6. Multiracial or Biracial 7. A race/ethnicity not listed here   Academic Rank   1. Instructor/Lecturer 2. Assistant Professor 3. Associate Professor 4. Professor   Do you have an administrative title?   1. Yes 2. No |
| **Burnout (n=2)** |
| *Select items from Maslach Burnout Inventory (MBI), based on previously published work: West CP, Dyrbye LN, Satele DV, et al. Concurrent validity of single-item measures of emotional exhaustion and depersonalization in burnout assessment. J Gen Intern Med. 2012;27:1445-1452. doi:10.1007/s11606-012-2015-7.:*  *Answer Never 0, Rarely 1, Sometimes 2, Frequently 3, Always 5*  Emotional Exhaustion  *“How often do you feel burned out from your work?”*  Depersonalization  *“How often do you feel you’ve become more callous toward people since you took this job?”*  *MBI - Human Services Survey for Medical Personnel - MBI-HSS (MP): Copyright ©1981, 2016 Christina Maslach & Susan E. Jackson. All rights reserved in all media. Published by Mind Garden, Inc., www.mindgarden.com*  **Note: While the full MBI-HSS survey was not utilized in this study, per the license to administer agreement and vendor Mind Garden: *The entire instrument form may not be included or reproduced at any time in any other published material.*** |
| **Personal Wellbeing (n=9)** |
| *Items from various forms (e.g. healthcare employee) of Well-Being Index (WBI)*  **Note: Per the license to administer agreement and Mayo Clinic Well-being Index :** *The Well-being Index instrument can be used for academic and IRB-approved research (which UNC received). In efforts to promote wellness research and validation, the Well-Being Index questions and scoring system are available for* ***free to qualifying users.*** *Per the WBI site,* ***“Please note:****You must* ***accept the terms and conditions of the Material Transfer Agreement regarding use of this intellectual property.”*** |
| **Professional Wellbeing (n=18)** |
| *Items from 18-item short version of MBI Areas of Worklife Survey (MBI-AWS):*  *Answer 1= Strongly agree, 2= Disagree, 3= Neither Agree nor Disagree, 4= Agree, 5= Strongly Agree*  Workload  Control  Reward  Community  Fairness  Values  *AWS Instrument - Copyright © 2000, 2011 by Michael P. Leiter & Christina Maslach. All rights reserved in all media. Published by Mind Garden, Inc., www.mindgarden.com*  **Per the license to administer agreement and vendor Mind Garden: *The entire instrument form may not be included or reproduced at any time in any other published material.*** |
| **Self-Efficacy (n=10)** |
| *Items adapted from General Self-Efficacy Scale (GSE):*  *Answer 1 = Not at all true   2 = Hardly true   3 = Moderately true   4 = Exactly true*   \| 1 \| I can always manage to solve difficult problems if I try hard enough. \| \| --- \| --- \| \| 2 \| If someone opposes me, I can find the means and ways to get what I want. \| \| 3 \| It is easy for me to stick to my aims and accomplish my goals. \| \| 4 \| I am confident that I could deal efficiently with unexpected events. \| \| 5 \| Thanks to my resourcefulness, I know how to handle unforeseen situations. \| \| 6 \| I can solve most problems if I invest the necessary effort. \| \| 7 \| I can remain calm when facing difficulties because I can rely on my coping abilities. \| \| 8 \| When I am confronted with a problem, I can usually find several solutions. \| \| 9 \| If I am in trouble, I can usually think of a solution. \| \| 10 \| I can usually handle whatever comes my way. \| |

**Career Advancement Questions** (to be added to Self-Efficacy well-being assessments)

In the last 12 months, have you experienced any of the following related to career advancements? (select all that apply)

- 1. Faculty reappointment
  2. Faculty promotion
  3. Change in tenure status
  4. Appointment to leadership position within your institution
  5. Appointment to leadership position for external professional organization/advisory board
  6. Other (open text)

In the last 12 months, have you had or received any of the following related to professional accomplishments? (select all that apply)

1. Peer-reviewed publication
2. Invited conference presentation
3. Grant and/or contract
4. Faculty teaching award
5. Faculty research award
6. Faculty service award
7. Other (open text)

In your opinion, what professional activity, accomplishment, or achievement has been the most rewarding in the last 12 months?

Short answer (open text)

Retention (answer yes/no)

In the last 12 months, have you moved to a new academic institution?

In the last 12 months, have you left your academic institution for a non-academic institution?

**Impact of Program on Well-being Questions (administered at conclusion of program):**

1. How has the “Well-being Initiative for Woman Faculty of Color” Program influenced, positively or negatively, your well-being?

2. What are 2-3 strategies that you feel you are most likely to apply to foster your personal and/or professional well-being?
