# Supplementary material for: Wellness in the invisible workforce: a pilot well-being study in black, indigenous, and people of color (BIPOC) women faculty in the pharmacy and pharmaceutical sciences
Source: BMC Med Educ. 2025 May 8;25:674. doi: 10.1186/s12909-025-07183-x (PMC12060405; doi:10.1186/s12909-025-07183-x)
Supplement: Supplementary file 2 — Supplementary Material 2: Additional file 2: Pre- and post-intervention summary statistics for primary and secondary outcomes by cohort. [file 12909_2025_7183_MOESM2_ESM.docx]

**Additional file 1. Pre- and post-intervention summary statistics for primary and secondary outcomes by cohort^^^**

| **Characteristic** | **Cohort 1^1^** | **Cohort 2^1^** |
| --- | --- | --- |
| Well-Being |  |  |
| Well-Being Index, Pre-intervention |  |  |
| Median (Min,Max) | 4.00 (0.00,6.00) | 3.00 (-1.00,7.00) |
| N Non-missing | 15 | 18 |
| At risk for low well-being, Pre-intervention | 14 / 15 (93%) | 14 / 18 (78%) |
| Well-Being Index, Post-intervention |  |  |
| Median (Min,Max) | 3.00 (-2.00,6.00) | 0.50 (-2.00,5.00) |
| N Non-missing | 11 | 6 |
| At risk for low well-being, Post-intervention | 8 / 11 (73%) | 1 / 6 (17%) |
| Professional Well-Being |  |  |
| Maslach AWS: Community Subscale, Pre-intervention |  |  |
| Median (Min,Max) | 2.67 (2.00,4.67) | 3.50 (1.67,5.00) |
| N Non-missing | 13 | 18 |
| Maslach AWS: Community Subscale, Post-intervention |  |  |
| Median (Min,Max) | 3.50 (2.00,4.33) | 3.67 (1.67,4.67) |
| N Non-missing | 10 | 6 |
| Maslach AWS: Reward Subscale, Pre-intervention |  |  |
| Median (Min,Max) | 2.67 (1.00,4.00) | 3.67 (1.33,5.00) |
| N Non-missing | 13 | 18 |
| Maslach AWS: Reward Subscale, Post-intervention |  |  |
| Median (Min,Max) | 3.83 (1.67,4.67) | 4.00 (2.67,4.67) |
| N Non-missing | 10 | 6 |
| Maslach AWS: Workload Subscale, Pre-intervention |  |  |
| Median (Min,Max) | 2.00 (1.00,3.00) | 2.83 (1.00,4.33) |
| N Non-missing | 13 | 18 |
| Maslach AWS: Workload Subscale, Post-intervention |  |  |
| Median (Min,Max) | 2.50 (1.00,3.33) | 2.50 (1.67,5.00) |
| N Non-missing | 10 | 6 |
| Maslach AWS: Control Subscale, Pre-intervention |  |  |
| Median (Min,Max) | 3.00 (1.00,4.33) | 3.83 (1.67,4.67) |
| N Non-missing | 13 | 18 |
| Maslach AWS: Control Subscale, Post-intervention |  |  |
| Median (Min,Max) | 3.33 (2.67,4.33) | 3.83 (3.00,5.00) |
| N Non-missing | 10 | 6 |
| Maslach AWS: Fairness Subscale, Pre-intervention |  |  |
| Median (Min,Max) | 2.33 (1.00,3.67) | 3.00 (1.00,3.67) |
| N Non-missing | 13 | 18 |
| Maslach AWS: Fairness Subscale, Post-intervention |  |  |
| Median (Min,Max) | 2.33 (1.33,3.33) | 2.83 (2.33,3.67) |
| N Non-missing | 10 | 6 |
| Maslach AWS: Values Subscale, Pre-intervention |  |  |
| Median (Min,Max) | 3.33 (3.00,3.67) | 3.17 (1.67,4.00) |
| N Non-missing | 13 | 18 |
| Maslach AWS: Values Subscale, Post-intervention |  |  |
| Median (Min,Max) | 3.50 (1.00,5.00) | 3.67 (3.00,4.00) |
| N Non-missing | 10 | 6 |
| Self-efficacy |  |  |
| General Self-Efficacy Survey, Pre-intervention |  |  |
| Median (Min,Max) | 31.00 (29.00,38.00) | 30.00 (23.00,34.00) |
| N Non-missing | 14 | 18 |
| General Self-Efficacy Survey, Post-intervention |  |  |
| Median (Min,Max) | 30.50 (30.00,34.00) | 34.00 (28.00,38.00) |
| N Non-missing | 6 | 6 |
| Burnout |  |  |
| Experience emotional or depersonalization burnout  at least once weekly, Pre-intervention | 7 / 11 (64%) | 11 / 18 (61%) |
| Experience emotional or depersonalization burnout  at least once weekly, Post-intervention | 4 / 10 (40%) | 2 / 6 (33%) |
| Experience emotional burnout  at least once weekly, Pre-intervention | 7 / 11 (64%) | 10 / 18 (56%) |
| Experience emotional burnout  at least once weekly, Post-intervention | 4 / 10 (40%) | 2 / 6 (33%) |
| Experience depersonalization burnout  at least once weekly, Pre-intervention | 1 / 11 (9.1%) | 2 / 18 (11%) |
| Experience depersonalization burnout  at least once weekly, Post-intervention | 1 / 10 (10%) | 0 / 6 (0%) |
| Secondary Outcomes:  Professional Accomplishments and Career Advancement |  |  |
| Moved to non-academic institution in past 12 months, Pre-intervention | 0 / 14 (0%) | 0 / 18 (0%) |
| Moved to non-academic institution in past 12 months, Post-intervention | 0 / 6 (0%) | 0 / 6 (0%) |
| Moved to a new academic institution in past 12 months, Pre-intervention | 1 / 14 (7.1%) | 5 / 18 (28%) |
| Moved to a new academic institution in past 12 months, Post-intervention | 0 / 5 (0%) | 1 / 6 (17%) |
| Faculty re-appointment in the past 12 months, Pre-intervention |  |  |
| Faculty reappointment | 6 / 14 (43%) | 5 / 5 (100%) |
| None reported | 8 / 14 (57%) | 0 / 5 (0%) |
| Faculty re-appointment in the past 12 months, Post-intervention |  |  |
| Faculty reappointment | 3 / 6 (50%) | 1 / 1 (100%) |
| None reported | 3 / 6 (50%) | 0 / 1 (0%) |
| Faculty promotion in the past 12 months, Pre-intervention |  |  |
| Faculty promotion | 2 / 14 (14%) | 4 / 4 (100%) |
| None reported | 12 / 14 (86%) | 0 / 4 (0%) |
| Faculty promotion in the past 12 months, Post-intervention |  |  |
| Faculty promotion | 1 / 6 (17%) | 1 / 1 (100%) |
| None reported | 5 / 6 (83%) | 0 / 1 (0%) |
| Change in tenure status in the past 12 months, Pre-intervention |  |  |
| Change in tenure status | 0 / 14 (0%) | 1 / 1 (100%) |
| None reported | 14 / 14 (100%) | 0 / 1 (0%) |
| Change in tenure status in the past 12 months, Post-intervention |  |  |
| Change in tenure status | 0 / 6 (0%) | 1 / 1 (100%) |
| None reported | 6 / 6 (100%) | 0 / 1 (0%) |
| Appointment to leadership position within your institution in pas 12 months, Pre-intervention |  |  |
| Appointment to leadership position within your institution | 2 / 14 (14%) | 4 / 4 (100%) |
| None reported | 12 / 14 (86%) | 0 / 4 (0%) |
| Appointment to leadership position within your institution in pas 12 months, Post-intervention |  |  |
| Appointment to leadership position within your institution | 1 / 6 (17%) | 2 / 2 (100%) |
| None reported | 5 / 6 (83%) | 0 / 2 (0%) |
| Appointment to leadership position for external professional organization/advisory board in past 12 months, Pre-intervention |  |  |
| Appointment to a leadership position for external professional organization/advisory board | 3 / 14 (21%) | 3 / 3 (100%) |
| None reported | 11 / 14 (79%) | 0 / 3 (0%) |
| Appointment to leadership position for external professional organization/advisory board in past 12 months, Post-intervention |  |  |
| Appointment to a leadership position for external professional organization/advisory board | 1 / 6 (17%) | 1 / 1 (100%) |
| None reported | 5 / 6 (83%) | 0 / 1 (0%) |
| Other career advancement in past 12 months, Pre-intervention |  |  |
| None reported | 12 / 14 (86%) | 0 / 1 (0%) |
| Other | 2 / 14 (14%) | 1 / 1 (100%) |
| Other career advancement in past 12 months, Post-intervention |  |  |
| None reported | 6 / 6 (100%) | 0 / 0 (NA%) |
| Peer-reviewed publication in past 12 months, Pre-intervention |  |  |
| None reported | 2 / 14 (14%) | 0 / 10 (0%) |
| Peer-reviewed publication | 12 / 14 (86%) | 10 / 10 (100%) |
| Peer-reviewed publication in past 12 months, Post-intervention |  |  |
| Peer-reviewed publication | 6 / 6 (100%) | 5 / 5 (100%) |
| Invited conference presentation in past 12 months, Pre-intervention |  |  |
| Invited conference presentation | 8 / 14 (57%) | 12 / 12 (100%) |
| None reported | 6 / 14 (43%) | 0 / 12 (0%) |
| Invited conference presentation in past 12 months, Post-intervention |  |  |
| Invited conference presentation | 3 / 6 (50%) | 2 / 2 (100%) |
| None reported | 3 / 6 (50%) | 0 / 2 (0%) |
| Grant and/or contract won in past 12 months, Pre-intervention |  |  |
| Grant and/or contract | 5 / 14 (36%) | 7 / 7 (100%) |
| None reported | 9 / 14 (64%) | 0 / 7 (0%) |
| Grant and/or contract won in past 12 months, Post-intervention |  |  |
| Grant and/or contract | 2 / 6 (33%) | 4 / 4 (100%) |
| None reported | 4 / 6 (67%) | 0 / 4 (0%) |
| Received faculty teaching award in past 12 months, Pre-intervention |  |  |
| Faculty teaching award | 3 / 14 (21%) | 4 / 4 (100%) |
| None reported | 11 / 14 (79%) | 0 / 4 (0%) |
| Received faculty teaching award in past 12 months, Post-intervention |  |  |
| Faculty teaching award | 2 / 6 (33%) | 0 / 0 (NA%) |
| None reported | 4 / 6 (67%) | 0 / 0 (NA%) |
| Received faculty research award in past 12 months, Pre-intervention |  |  |
| Faculty research award | 3 / 14 (21%) | 0 / 0 (NA%) |
| None reported | 11 / 14 (79%) | 0 / 0 (NA%) |
| Received faculty research award in past 12 months, Post-intervention |  |  |
| Faculty research award | 1 / 6 (17%) | 2 / 2 (100%) |
| None reported | 5 / 6 (83%) | 0 / 2 (0%) |
| Received faculty service award in past 12 months, Pre-intervention |  |  |
| Faculty service award | 2 / 14 (14%) | 1 / 1 (100%) |
| None reported | 12 / 14 (86%) | 0 / 1 (0%) |
| Received faculty service award in past 12 months, Post-intervention |  |  |
| Faculty service award | 1 / 6 (17%) | 2 / 2 (100%) |
| None reported | 5 / 6 (83%) | 0 / 2 (0%) |
| Other professional accomplishment in past 12 months, Pre-intervention |  |  |
| None reported | 12 / 14 (86%) | 0 / 2 (0%) |
| Other | 2 / 14 (14%) | 2 / 2 (100%) |
| Other professional accomplishment in past 12 months, Post-intervention |  |  |
| None reported | 5 / 6 (83%) | 0 / 0 (NA%) |
| Other | 1 / 6 (17%) | 0 / 0 (NA%) |
| ^1^n / N Non-missing (%)  ^ Each assessment for each cohort was summarized using all the study participants who completed that assessment, thus sample sizes varied among assessments. Consistent with goals of a pilot study being preliminary estimation of effect size, as well as the small sample size, p-values were not provided. | | |
